# Extracellular vesicles are dynamic regulators of maternal glucose homeostasis during pregnancy

**DOI:** 10.1038/s41598-023-31425-x

**Published:** 2023-03-20

**Authors:** Hannah C. Zierden, Ruth Marx-Rattner, Kylie D. Rock, Kristen R. Montgomery, Pavlos Anastasiadis, Lillian Folts, Tracy L. Bale

**Affiliations:** 1 Department of Pharmacology, University of Maryland School of Medicine, Baltimore, MD 21201 USA; 2 Center for Epigenetic Research in Child Health and Brain Development, University of Maryland School of Medicine, Baltimore, MD 21201 USA; 3 Department of Neurosurgery, University of Maryland School of Medicine, Baltimore, MD 21201 USA; 4 Marlene and Stewart Greenebaum Comprehensive Cancer Center, University of Maryland, Baltimore, MD 21201 USA; 5 Present Address: The Anschutz Foundation Endowed Chair in Women’s Integrated Mental and Physical Health Research at the Ludeman Center, Aurora, CO USA; 6 Present Address: Department of Psychiatry, University of Colorado School of Medicine, CU Anschutz Medical Campus, 12800 E. 19th Avenue, Aurora, CO 80045 USA; 7 Present Address: Department of Chemical and Biomolecular Engineering, University of Maryland, College Park, MD 20740 USA; 8 Present Address: Department of Biological Sciences, North Carolina State University, Raleigh, NC 27695 USA; 9 Present Address: Neuroscience Graduate Program, University of Colorado Anschutz Medical Campus, Aurora, CO 80045 USA; 10 Present Address: Biomedical Sciences Graduate Program, University of Colorado Anschutz Medical Campus, Aurora, CO 80045 USA

**Keywords:** Predictive markers, Extracellular signalling molecules, Metabolic disorders

## Abstract

Homeostatic regulation of the maternal milieu during pregnancy is critical for maternal and fetal health. The placenta facilitates critical communication between maternal and fetal compartments, in part, through the production of extracellular vesicles (EVs). EVs enable tissue synchrony via cell–cell and long-distance communication and are at their highest circulating concentration during pregnancy. While much work has been done investigating how physiological challenges in pregnancy affect the fetus, the role of placental communication in maternal health has not been well examined. We previously identified placental O-glycosyl transferase (OGT), a glucose-sensing enzyme, as a target of maternal stress where OGT levels and activity affected the O-glycosylation of proteins critical for EV cargo loading and secretion. Here, we hypothesized that placental OGT plays an essential role in maternal homeostatic regulation during pregnancy via its regulation of maternal circulating EV concentrations. Our studies found that changes to key metabolic factors over the circadian cycle, including glucocorticoids, insulin, and glucose, were significantly associated with changes in circulating EV concentration. Targeting placental OGT in mice, we found a novel significant positive relationship between placental OGT and maternal circulating EV concentration that was associated with improving maternal glucose tolerance during pregnancy. Finally, an intravenous elevation in EVs, matching the concentration of EVs during pregnancy, shifted non-pregnant female glucose sensitivity, blunted glucose variance, and improved synchrony of glucose uptake. These data suggest an important and novel role for circulating EVs as homeostatic regulators important in maternal health during pregnancy.

## Introduction

Disruptions to homeostasis during pregnancy, including chronic stress, are associated with adverse maternal health outcomes including preterm birth, gestational diabetes, and preeclampsia^1–5^. In our prior studies, we found that placental levels of the X-linked gene and glucose-sensing enzyme, O-glycosyl transferase (OGT), were directly related to sex-specific offspring outcomes resulting from maternal stress^6–8^. OGT serves as a link between maternal glucose regulation and placental function during pregnancy^9^. As the placenta reflects the fetal chromosomal sex, in both mouse and human placentae, females have twice as much OGT protein and enzymatic activity (O-GlcNAcylation) as males^10–13^. These higher levels seem to protect the female fetus from maternal insults, including stress^14^. However, despite these extensive mechanistic studies examining the importance of the placenta in fetal development, its role in maternal health has not been well examined.

A key function of the placenta in the maintenance of pregnancy is cellular communication and coordination between fetal and maternal compartments^15–20^. A primary mechanism of this short- and long-distance communication is signaling via extracellular vesicles (EVs)^21,22^. EVs are biologically produced nanoparticles that transport proteins, nucleic acids, and small molecules to coordinate physiologic functions^22–24^. Circulating EVs are a dynamic and heterogeneous population of various sized particles (macro-vesicles > micro-vesicles > exosomes) from different cellular origins with diverse functions^25,26^. EVs produced during pregnancy are involved in implantation, immune activation, angiogenesis, glucose metabolism and uptake, and the onset of labor^27–31^. During pregnancy, the concentration of EVs in maternal circulation is dramatically increased, 3–fourfold, which is attributed to the large population of EVs produced by the placenta, supporting the hypothesis that placental EVs are involved in maternal homeostatic regulation in pregnancy^21,31–33^. In fact, changes in EV cargo and secretion associated with gestational diabetes have been well characterized across pregnancy suggesting their potential role to counter homeostatic imbalance^34–38^. Furthermore, we previously observed significant changes to O-GlcNAcylation of placental Annexin A1 (ANXA1), a protein involved in EV secretion, as a result of maternal stress during pregnancy, leading us to investigate the molecular mechanisms involved in how placental OGT acts as regulator of EV secretion into maternal circulation^6,39^. As our previous studies demonstrated a significant reduction of OGT by maternal stress, we first examine the effect of stress to alter EV secretion in both the nonpregnant and pregnant state. We then focus on the specific role of OGT to significantly regulate EV concentration, and finally the ability for an increase in pregnancy EVs to affect glucose homeostasis.

## Results

### Circulating extracellular vesicle (EV) concentrations significantly change in response to altered metabolic state.

**Figure 1:**
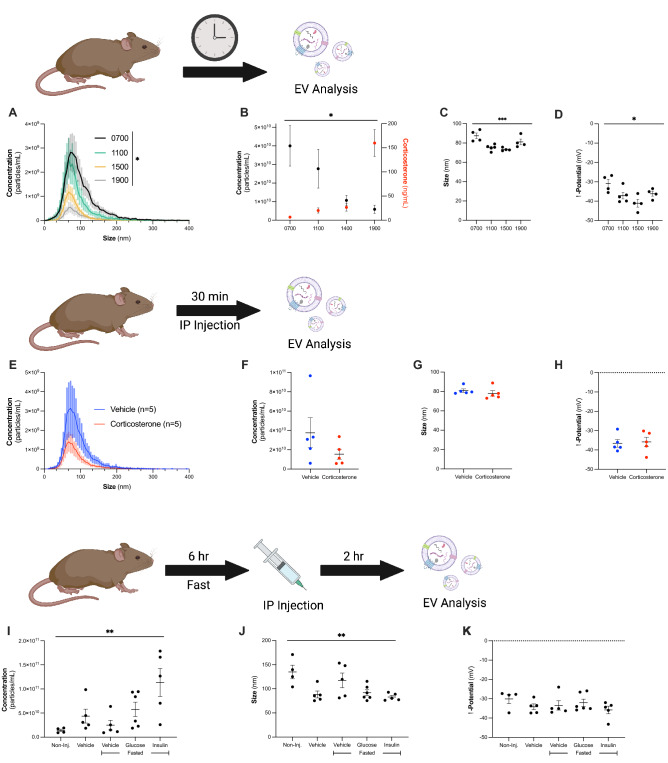
Circulating extracellular vesicle (EV) concentrations significantly change in response to altered metabolic state. (**A**) EV concentration in circulation significantly changes over the course of the day (one-way ANOVA, main effect of time, F_3,13_ = 3.509, *p* = 0.0463). 0700 shown in black, n = 4; 1100 in green, n = 5; 1500 in yellow, n = 4; 1900 in gray, n = 4. (**B**) The concentration of circulating EVs decreases over the course of the day (left y-axis, black, one-way ANOVA, main effect of time, F_3,13_ = 3.509, *p* = 0.0463), while corticosterone levels increase over the course of the day (right y-axis, red, one-way ANOVA, main effect of time, F_3,13_ = 25.62, *p* < 0.0001). (**C**) The size of circulating EVs significantly changes over the course of the circadian rhythm (one-way ANOVA, main effect of time, F_3,13_ = 8.454, *p* = 0.0023), and (**D**) the ζ-potential is significantly changed (one-way ANOVA, main effect of time, F_3,13_ = 5.023, *p* = 0.0158). (**E**,**F**) IP injections of corticosterone (red, n = 5) trend to decrease EV concentration in non-pregnant dams as compared to vehicle injections (blue, n = 5, two-tailed *t*-test, *t*_*8*_ = 1.351, *p* = 0.2137). Corticosterone injections did not impact (**G**) size (two-tailed *t*-test, *t*_*8*_ = 1.351, *p* = 0.2137) or (**H**) ζ-potential of circulating EVs (two-tailed *t*-test, *t*_*8*_ = 0.2309, *p* = 0.8232). (**I**) Non-pregnant dams received either no injection (n = 4), vehicle injection (saline, n = 5), 6 h fasting with a vehicle injection (n = 5), 6 h fasting with a glucose injection (3 mg glucose/g body weight, n = 6), or 6 h fasting with an insulin injection (0.8 mU insulin/g body weight, n = 5). There was a significant effect of injection on concentration (one-way ANOVA, main effect of treatment, F_4,20_ = 4.883, *p* = 0.0065) and (**J**) size (one-way ANOVA, main effect of treatment, F_4,20_ = 4.764, *p* = 0.0073) of circulating EVs but not on (**K**) ζ-potential of circulating EVs.

We first compared how the circadian rhythm affects circulating EVs in comparison with corticosterone levels over the course of the day. The concentration of circulating EVs significantly decreased (one-way ANOVA, main effect of time, F_3,13_ = 3.509, *p* = 0.0463) while the concentration of corticosterone significantly increased (Fig. 1A–D, one-way ANOVA, main effect of time, F_3,13_ = 25.62, *p* < 0.0001). While not significantly associated with exogenous changes to corticosterone, the size of EVs was significantly changed over the course of the day (Fig. 1C, one-way ANOVA, main effect of time, F_3,13_ = 8.454, *p* = 0.0023), with significant differences between 0700 h (87.5 ± 2.9) and 1100 h (75.2 ± 1.4, *p* = 0.006), and 0700 h and 1500 h (73.4 ± 0.803, *p* = 0.0031). The ζ-potential of EVs also changed over the course of the day (Fig. 1D, one-way ANOVA, main effect of time, F_3,13_ = 5.023, *p* = 0.0158), with EVs at 0700 h (− 30.9 ± 2.2 mV) being significantly more neutral than EVs at 1500 h (− 41.1 ± 2.0 mV, *p* = 0.0096).

We next sought to probe the direct effect of the stress hormone, corticosterone, on circulating EVs using non-pregnant dams. We injected non-pregnant dams with vehicle (saline, n = 5) or corticosterone (300 μg/kg, n = 5) and evaluated the concentration of circulating EVs at 30 min. The vehicle group had an average concentration of 3.7 × 10^10^ ± 1.5 × 10^10^ particles/mL, while the corticosterone treated group had a concentration of 1.5 × 10^10^ ± 5.2 × 10^9^ particles/mL (Fig. 1E–H, n = 5 per group). While not statistically significant, there was a trend towards decreased EV concentration with increasing corticosterone treatment (Fig. 1F, two-tailed *t*-test, *t*_*8*_ = 1.351, *p* = 0.2137). We observed no differences in either size (Fig. 1G, two-tailed *t*-test, *t*_*8*_ = 0.8502, *p* = 0.4200) or ζ-potential (Fig. 1H, two-tailed *t*-test, *t*_*8*_ = 0.2309, *p* = 0.8232) of EVs associated with corticosterone treatment. EVs from the vehicle group were 80.9 ± 1.8 nm, with a ζ-potential of -36.6 ± 1.9 mV, while EVs from corticosterone treated dams were 78.1 ± 2.8 nm, with a ζ-potential of − 35.9 ± 2.5 nm.

To further examine homeostatic factors that change in response to stress and circadian rhythm, we examined the effect of fasting (n = 5), a glucose bolus injection (n = 6), and an insulin bolus injection (n = 5) on EV concentration in non-pregnant dams. As a comparison for the stress of the injection, non-injected dams (n = 4) averaged an EV concentration of 1.4 × 10^10^ ± 2.5 × 10^9^ particles/mL, while the vehicle injected group (saline, n = 5) averaged 4.4 × 10^10^ ± 1.4 × 10^10^ particles/mL, fasting vehicle injected group averaged 2.5 × 10^10^ ± 1.0 × 10^10^ particles/mL, fasting glucose injected group averaged 5.8 × 10^10^ ± 1.5 × 10^10^ particles/mL (Fig. 1I), and fasting insulin injected group averaged 1.1 × 10^11^ ± 2.9 × 10^10^ particles/mL. The concentration of EVs in circulation significantly changed across treatment (one-way ANOVA, main effect of treatment, F_4,20_ = 4.883, *p* = 0.0065), where the insulin injection showed a significant increase in EV concentration compared to the non-injected and fasted groups (*p* = 0.0078, and *p* = 0.0125, respectively). The non-injected group had an average size of 135.0 ± 14.1 nm, vehicle 88.4 ± 7.1 nm, fasting 117.6 ± 15.1 nm, glucose 91.9 ± 6.1 nm, and insulin 83.1 ± 3.3 nm (Fig. 1J). We observed significant differences in the size of particles across these groups (one-way ANOVA, main effect of treatment, F_4,20_ = 4.764, *p* = 0.0073), where EVs from the non-injected group were significantly larger than EVs from vehicle (*p* = 0.0311), glucose (*p* = 0.041), and insulin (*p* = 0.014) injected groups. There were no significant differences in the ζ-potential of EVs across treatments, with the EVs from the non-injected group having a ζ-potential of − 30.1 ± 2.4 mV, vehicle − 34.2 ± 1.5 mV, fasting − 33.5 ± 2.4 mV, glucose − 32.1 ± 1.9 mV, and insulin − 33.5 ± 2.0 mV (Fig. 1K). These data demonstrate that EV production relies on many compounding factors, and we next used our mouse model of early pregnancy stress to understand the potential role of EVs in controlling homeostatic changes during pregnancy.

### Stress during pregnancy significantly decreases circulating EV concentration

**Figure 2:**
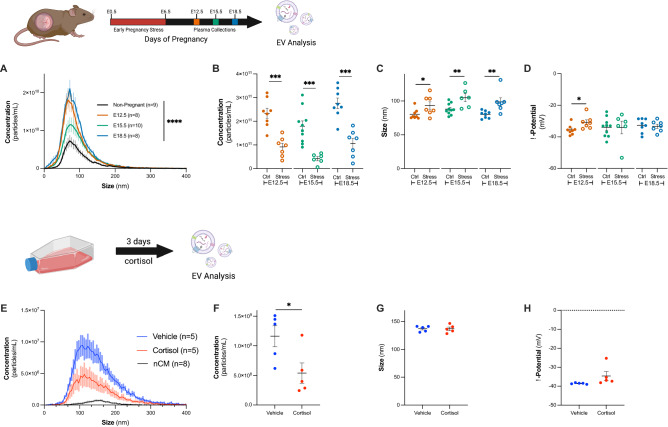
Robust increase in circulating EV concentration during pregnancy is significantly decreased by stress in vivo, and glucocorticoids in vitro. (**A**) There was a significant effect of pregnancy on the concentration of EVs in vivo (one-way ANOVA, main effect of treatment, F_3,31_ = 12.46, *p* < 0.0001). Non-pregnant dams shown in black (n = 9), E12.5 dams in orange (n = 8), E15.5 dams in green (n = 10), and E18.5 dams in blue (n = 8). (**B**–**D**) We investigated changes to circulating EVs as a result of maternal stress (open circles). (**B**) E12.5 stressed dams (n = 7, open orange circles) had a concentration of 9.1 × 10^10^ ± 1.6 × 10^10^ particles/mL plasma, at E15.5 (n = 6, open green circles) 4.2 × 10^10^ ± 8.6 × 10^9^ particles/mL, and at E18.5 (n = 7, open blue circles) 1.1 × 10^11^ ± 2.2 × 10^10^ particles/mL. Stress produced a significant decrease in circulating EVs (E12.5, two-tailed *t*-test, *t*_*13*_ = 5.285, *p* = 0.0001; E15.5, two-tailed *t*-test, *t*_*14*_ = 4.502, *p* = 0.0005; E18.5, two-tailed *t*-test, *t*_*13*_ = 5.424, *p* = 0.0001) and (**C**) a significant increase in EV size. EVs from E12.5 control dams were 80.38 ± 2.7 nm, whereas EVs from stressed dams were 93.3 ± 5.5 nm (two-tailed *t*-test, *t*_*13*_ = 2.205, *p* = 0.046). EVs from E15.5 control dams were 88.0 ± 2.6 nm, whereas EVs from stressed dams were 104.8 ± 5.8 nm (two-tailed *t*-test, *t*_*14*_ = 3.028, *p* = 0.009). EVs from E18.5 control dams were 80.1 ± 1.9 nm, and EVs from E18.5 stressed dams were 98.7 ± 5.9 nm (two-tailed *t*-test, *t*_*13*_ = 3.143, *p* = 0.0078). (**D**) E12.5 EVs from control dams were − 35.5 ± 1.1 mV, and E12.5 EVs from stressed dams were − 30.8 ± 1.6 mV (two-tailed *t*-test, *t*_*13*_ = 2.503, *p* = 0.0264). E15.5 EVs from control dams were − 34.0 ± 1.9 mV, and E15.5 EVs from stressed dams were − 34.1 ± 1.6 mV (two-tailed *t*-test, *t*_*14*_ = 0.03167, *p* = 0.9752). E18.5 EVs from control dams were − 32.7 ± 1.6 mV, and E18.5 EVs from stressed dams were − 33.3 ± 1.3 mV (two-tailed *t*-test, *t*_*13*_ = 0.2837, *p* = 0.7811). (**E**) Cortisol treatment (red, n = 5) significantly decreased concentration of EVs in media of BeWo-b30 cells to 5.39 × 10^8^ ± 1.71 × 10^8^ particles/mL, compared to vehicle treated (blue, n = 5) which had a concentration of from 1.16 × 10^9^ ± 1.77 × 10^8^ particles/mL. Non-conditioned media (black, n = 8) had a concentration of 6.54 × 10^7^ ± 4.89 × 10^6^ particles/mL. (**F**) The concentration of EVs secreted into the media was significantly decreased following cortisol treatment (two-tailed *t*-test, *t*_*8*_ = 2.532, *p* = 0.0351). Cortisol exposure did not alter the (**G**) size (vehicle = 137.0 ± 3.1 nm v. cortisol = 137.1 ± 2.2 nm, two-tailed *t*-test, *t*_*8*_ = 0.0275, *p* = 0.9787) or (**H**) ζ-potential of EVs (vehicle = -34.6 ± 2.4 mV v. cortisol = − 38.5 ± 0.15 mV, two-tailed *t*-test, *t*_*8*_ = 1.652, *p* = 0.1372).

We first explored changes in circulating vesicles over the course of normal pregnancy, specifically on E12.5, E15.5 and E18.5, where plasma was collected from pregnant dams and used to isolate EVs. As expected, we observed a significant increase in the concentration of circulating EVs over the course of pregnancy (Fig. 2A, one-way ANOVA, main effect of treatment, F_3,31_ = 12.46, *p* < 0.0001). The concentration of EVs from non-pregnant dams (1.0 × 10^11^ ± 1.8 × 10^10^ particles/mL, n = 9) was significantly lower than EVs from pregnant dams at all timepoints (E12.5, 2.3 × 10^11^ ± 2.1 × 10^10^ particles/mL, *p* = 0.0007, n = 8; E15.5, 1.8 × 10^11^ ± 2.3 × 10^10^ particles/mL, *p* = 0.0489, n = 10; E18.5, 2.8 × 10^11^ ± 2.2 × 10^10^ particles/mL, *p* < 0.0001, n = 8).

As we previously identified changes to O-GlcNAcylation of placental ANXA1 as a result of maternal stress during pregnancy^6^, we next evaluated changes to circulating EVs following maternal stress (Fig. 2B–D). At E12.5 stressed dams (n = 7) had a concentration of 9.1 × 10^10^ ± 1.6 × 10^10^ particles/mL plasma, at E15.5 (n = 6) 4.2 × 10^10^ ± 8.6 × 10^9^ particles/mL, and at E18.5 (n = 7) 1.1 × 10^11^ ± 2.2 × 10^10^ particles/mL (Fig. 2B). Across all three gestational days, there was a significant decrease in concentration of EVs associated with stress (Fig. 2B, E12.5, two-tailed *t*-test, *t*_*13*_ = 5.285, *p* = 0.0001; E15.5, two-tailed *t*-test, *t*_*14*_ = 4.502, *p* = 0.0005; E18.5, two-tailed *t*-test, *t*_*13*_ = 5.424, *p* = 0.0001). We also compared the size and ζ-potential of particles in each group. We observed an increase in particle size associated with stress (Fig. 2C). At E12.5, EVs from control dams were 80.38 ± 2.7 nm, whereas EVs from stressed dams were 93.3 ± 5.5 nm (two-tailed *t*-test, *t*_*13*_ = 2.205, *p* = 0.046). EVs from control dams at E15.5 were 88.0 ± 2.6 nm, whereas EVs from stressed dams showed an increase in size to 104.8 ± 5.8 nm (two-tailed *t*-test, *t*_*14*_ = 3.028, *p* = 0.009). Similarly, at E18.5 EVs from the control group were 80.1 ± 1.9 nm, and those from the stressed group were 98.7 ± 5.9 nm (two-tailed *t*-test, *t*_*13*_ = 3.143, *p* = 0.0078). We only observed significant differences in the ζ-potential of circulating EVs on E12.5 (Fig. 2D). On E12.5, EVs from control dams were − 35.5 ± 1.1 mV, whereas EVs from stressed dams were − 30.8 ± 1.6 mV (two-tailed *t*-test, *t*_*13*_ = 2.503, *p* = 0.0264). EVs from control dams at E15.5 were − 34.0 ± 1.9 mV, and EVs from stressed dams were − 34.1 ± 1.6 mV (two-tailed *t*-test, *t*_*14*_ = 0.03167, *p* = 0.9752). On E18.5, EVs from the control group were − 32.7 ± 1.6 mV, and those from the stressed group were − 33.3 ± 1.3 mV (two-tailed *t*-test, *t*_*13*_ = 0.2837, *p* = 0.7811). As previously reported, we found no difference in the litter size between control and stressed treatment groups (Supplemental Fig. 1A).

In an in vitro model using a pure population of human trophoblast BeWo b30 cells, we investigated the effect of the stress hormone, cortisol, on EV production. Again, we saw a significant reduction in EV secretion in response to cortisol where vehicle treatment had an average concentration of 1.2 × 10^9^ ± 1.8 × 10^8^ particles/mL (n = 5), while the cortisol treated group had a reduction in concentration to 5.4 × 10^8^ ± 1.7 × 10^8^ particles/mL (Fig. 2E,F, n = 5). The non-conditioned media had a concentration of 6.5 × 10^7^ ± 4.9 × 10^6^ particles/mL (n = 8). As in other stress models, we observe that stress hormone significantly decreased EV concentration in this pure population of cells (two-tailed *t*-test, *t*_*8*_ = 2.532, *p* = 0.0351). We observed no difference in size (Fig. 2G, vehicle = 137.0 ± 3.1 nm v. cortisol = 137.1 ± 2.2 nm, two-tailed *t*-test, *t*_*8*_ = 0.0275, *p* = 0.9787) or ζ-potential (Fig. 2H, vehicle = − 34.6 ± 2.4 mV v. cortisol = − 38.5 ± 0.15 mV, two-tailed *t*-test, *t*_*8*_ = 1.652, *p* = 0.1372) for the two groups.

### Increasing circulating EV concentration shifts glucose dynamics

**Figure 3:**
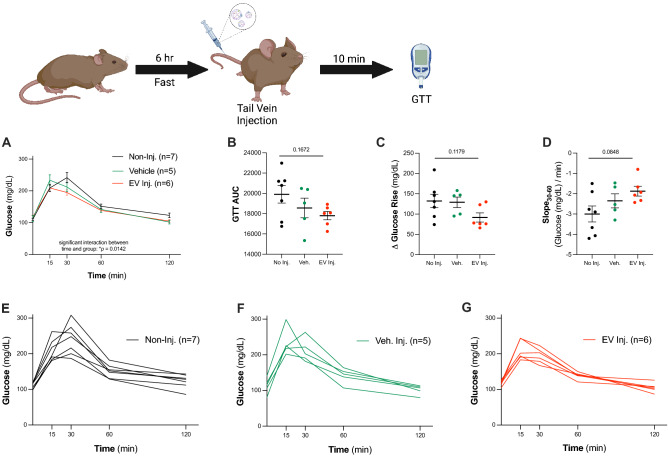
Dynamic increase of circulating EV concentration shifts glucose dynamics. (**A**) Glucose tolerance curves for non-pregnant dams that received no injection (n = 7, black), vehicle injection (n = 5, green), or EV injection (n = 6, red) (two-way ANOVA, main effect of time, F_2.61,39.14_ = 122.7, *p* < 0.0001, main effect of injection, F_2,15_ = 1.265, *p* = 0.3106, time*injection interaction, F_8,60_ = 2.666, *p* = 0.0142). There was no significant difference in the glucose tolerance test (**B**) area under the curve (one-way ANOVA, main effect of injection, F_2,15_ = 2.02, *p* = 0.1672, Bartlett’s test, *p* = 0.2105, SD_NoInj_ = 2296, SD_VehInj_ = 2162, SD_EVInj_ = 1005), (**C**) change in glucose from 0 to 15 min (one-way ANOVA, main effect of injection, F_2,15_ = 2.474, *p* = 0.1179, Bartlett’s test, *p* = 0.5110, SD_NoInj_ = 44.05, SD_VehInj_ = 29.23, SD_EVInj_ = 27.52), or (**D**) slope of glucose processing from 30 to 60 min (one-way ANOVA, main effect of injection, F_2,15_ = 2.922, *p* = 0.0848, Bartlett’s test, *p* = 0.4621, SD_NoInj_ = 1.045, SD_VehInj_ = 0.7723, SD_EVInj_ = 0.5935). (**E–G**) Individual dam glucose tolerance curves are shown (**E**) for non-injected dams, (**F**) vehicle injected dams, and (**G**) EV injected dams.

Pregnancy is a highly metabolic and physiologically demanding state. Using glucose tolerance tests (GTT), clinical and preclinical studies previously demonstrated that glucose sensitivity is diminished as pregnancy progresses^40^. As we previously identified placental O-GlcNAc transferase (OGT), a nutrient sensing enzyme that drives the sex-specific effects of maternal stress on offspring, we sought to examine glucose uptake and processing as a readout of the role of EVs in maternal metabolic health^6,7,41^. We first tested the hypothesis that acutely increasing the concentration of EVs in circulation by injecting EVs from E18.5 pregnant dams into the tail vein of non-pregnant dams would impact glucose sensitivity and processing (Fig. 3). We observed significant differences in glucose tolerance between non-injected (n = 7), vehicle (saline, n = 5), and EV-injected treatment (Fig. 3A, n = 6, two-way ANOVA, main effect of time, F_2.61,39.14_ = 122.7, *p* < 0.0001, main effect of injection, F_2,15_ = 1.265, *p* = 0.3106, time*injection interaction, F_8,60_ = 2.666, *p* = 0.0142). While we observed no statistically significant differences when analyzing glucose tolerance, we consistently observed glucose rise and slope recovery patterns suggesting that an increased EV concentration affects glucose processing. Increasing circulating EVs trended towards decreasing the area under the curve (AUC) (Fig. 3B, one-way ANOVA, main effect of injection, F_2,15_ = 2.02, *p* = 0.1672, Bartlett’s test, *p* = 0.2105, SD_NoInj_ = 2296, SD_VehInj_ = 2162, SD_EVInj_ = 1005), decreasing the change in glucose rise from 0 to 15 min (Fig. 3C, one-way ANOVA, main effect of injection, F_2,15_ = 2.474, *p* = 0.1179, Bartlett’s test, *p* = 0.5110, SD_NoInj_ = 44.05, SD_VehInj_ = 29.23, SD_EVInj_ = 27.52), and increasing glucose processing, as indicated by an increased slope of the curve from 30 to 60 min (Fig. 3D, one-way ANOVA, main effect of injection, F_2,15_ = 2.922, *p* = 0.0848, Bartlett’s test, *p* = 0.4621, SD_NoInj_ = 1.045, SD_VehInj_ = 0.7723, SD_EVInj_ = 0.5935). Overall, acutely increasing EV concentration trended toward a synchronization of glucose response across individual animals, as indicated by a reduced group variance (Fig. 3E–G). These data, while only patterns and not statistically significant, suggest that circulating EVs may play a role in coordinating glucose sensitivity and uptake, a critical biologic process during pregnancy.

### O-glycosyl transferase (OGT) levels in the placenta affect circulating EVs

OGT serves as a link between maternal glucose regulation and placental function during pregnancy^9^. The placenta contributes to the significant increase in circulating EVs during pregnancy. Therefore, we utilized genetic targeting of placental OGT to determine its contribution to overall circulating EVs. As mice have litters and, therefore, multiple placentae, we developed a scoring system to compare the amount of OGT in the uterus between dams, where each copy of OGT in the placenta received 1 point (X^WT^/X^WT^ = 2 points; X^OGT−^/X^WT^ or X^WT^/Y = 1 point; X^OGT−^/X^OGT−^ or X^OGT−^/Y = 0 points). As with stress, altered OGT expression in the placenta did not affect litter size (Supplementary Fig. 1B).

**Figure 4:**
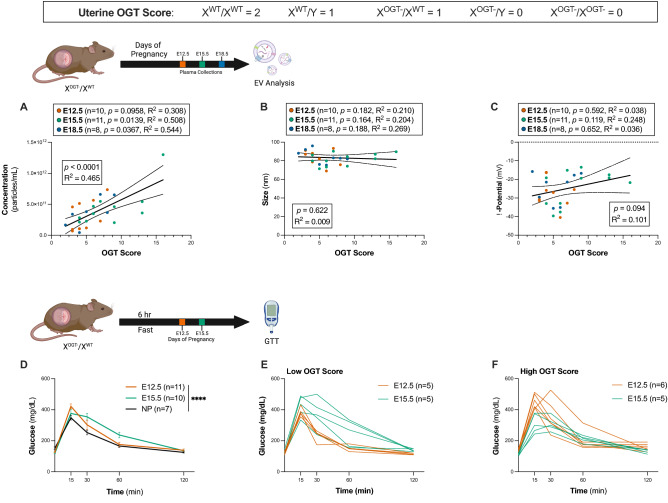
Placental OGT levels significantly correlate with circulating EV concentration and maternal glucose sensitivity. (**A**) Across pregnancy there was a significant correlation between OGT score and concentration of EVs (Pearson correlation, F_1,27_ = 23.43, *p* < 0.0001, R^2^ = 0.4646). The concentration EVs on E12.5 (n = 10, orange circles) was not significantly correlated with OGT score (Pearson correlation, F_1,8_ = 3.562, *p* = 0.0958, R^2^ = 0.308). The concentration of EVs from E15.5 dams (n = 11, green circles) was significantly with OGT score (Pearson correlation, F_1,9_ = 9.286, *p* = 0.0139, R^2^ = 0.508) and the concentration of EVs from E18.5 dams (n = 8, green circles) was significantly correlated with OGT score (Pearson correlation, F_1,6_ = 7.159, *p* = 0.0367, R^2^ = 0.544). 95% CI are shown as dotted lines above and below the trend line. (**B**) We observed no correlation for the size of circulating EVs with uterine OGT score. Similarly, no trends were observed for circulating EVs on E12.5 (n = 10, orange, Pearson correlation, F_1,8_ = 2.131, *p* = 0.182, R^2^ = 0.2104), E15.5 (n = 11, green, Pearson correlation, F_1,9_ = 2.3, *p* = 0.164, R^2^ = 0.2036), or E18.5 (n = 8, blue, Pearson correlation, F_1,6_ = 2.207, *p* = 0.188, R^2^ = 0.2689) (**C**) Across pregnancy, the ζ-potential of EVs from X^OGT−^/X^WT^ dams did not significantly change with OGT score (Pearson correlation, F_1,27_ = 3.017, *p* = 0.094, R^2^ = 0.1005). There was no change in the ζ-potential of EVs on E12.5 (n = 10, orange, Pearson correlation, F_1,8_ = 0.3123, *p* = 0.592, R^2^ = 0.0376), E15.5 (n = 11, green, Pearson correlation, F_1,9_ = 2.971, *p* = 0.119, R^2^ = 0.2482), or E18.5 (n = 8, blue, Pearson correlation, F_1,6_ = 0.2253, *p* = 0.652, R^2^ = 0.0362). 95% CI are shown as dotted lines above and below trend lines. (**D**) Glucose tolerance for non-pregnant (n = 7, NP, black), E12.5 (n = 11, orange) and E15.5 (n = 10, green) dams. We observed significant variation in glucose sensitivity across gestational days (two-way ANOVA, main effect of time, F_2.324,58.11_ = 187.8, *p* < 0.0001, main effect of group, F_2,25_ = 2.241, *p* = 0.1273, time*group interaction, F_8,100_ = 5.310, *p* < 0.0001). (**E**) Glucose tolerance curves for pregnant dams with a low (< 7) OGT score. Individual E12.5 dams are shown in orange (n = 5) and E15.5 in green (n = 5). (**F**) Glucose tolerance curves for pregnant dams with a high (≥ 7) OGT score. Individual E12.5 dams shown in orange (n = 6) and E15.5 in green (n = 5).

We found a significant positive correlation between the overall uterine OGT score and the concentration of EVs in maternal circulation (Fig. 4A, Pearson correlation, F_1,27_ = 23.43,* p* < 0.0001, R^2^ = 0.4646). Examining these data by gestational day, we found that the concentration of EVs at E12.5 (n = 10, Pearson correlation, F_1,8_ = 3.562, *p* = 0.0958, R^2^ = 0.308), E15.5 (n = 11, Pearson correlation, F_1,9_ = 9.286, *p* = 0.0139, R^2^ = 0.508), and E18.5 (n = 8, Pearson correlation, F_1,6_ = 7.159, *p* = 0.0367, R^2^ = 0.544) were correlated with overall uterine OGT score, with a statistically significant relationship between increasing uterine OGT and increased maternal circulating EV concentration. We observed no association between either particle size and OGT score (Fig. 4B), or between ζ-potential and OGT score (Fig. 4C). Importantly, we demonstrate that this is driven by OGT and not by the number of placentae (Supplementary Fig. 2). While OGT score is significantly correlated to the number of placentae (Supplementary Fig. 2A, Pearson correlation, F_1,27_ = 13.67, *p* = 0.001, R^2^ = 0.3361), when restricting the data analysis to include only litters of 7–8 pups, we observed a wide range of OGT scores (2–7, Supplementary Fig. 2B). Similarly, when restricting the analysis to include only litters of 7–8 pups, we observed a trend in increasing EV concentration with OGT score (Supplementary Fig. 2C, Pearson correlation, F_1,10_ = 2.279, *p* = 0.162, R^2^ = 0.1856).

### Levels of placental OGT significantly correlate with circulating EV concentration and maternal glucose sensitivity

We next examined the potential relationship between placental OGT and maternal glucose tolerance over the course of pregnancy. As observed in clinical and preclinical studies, we found a significant reduction in glucose sensitivity as pregnancy progressed (two-way ANOVA, main effect of time, F_2.324,58.11_ = 187.8, *p* < 0.0001, main effect of group, F_2,25_ = 2.241, *p* = 0.1273, time*group interaction, F_8,100_ = 5.310, *p* < 0.0001) (Fig. 4D). Further linking placental OGT and EV secretion with glucose regulation, we then investigated the association of the uterine OGT score (as above) with maternal glucose tolerance. Based on the average OGT score across all pregnancies, each dam was characterized as having a low or high total uterine OGT score, (n = 58, Supplementary Fig. 1C). At the mid-gestational timepoint, E12.5, prior to the onset of glucose insensitivity in pregnancy, dams with an OGT score < 7 showed more efficient glucose processing (n = 5, Fig. 4E). However, by E15.5 dams with an OGT ≥ 7 showed more efficient glucose processing (n = 5, Fig. 4F). At the end of pregnancy, on E18.5, dams with an OGT ≥ 7 showed more efficient glucose processing (n = 6, Supplementary  Fig. 3). The high variability we observed at the end of pregnancy may be attributed to the impending homeostatic changes related to parturition. As maternal glucose sensitivity is reduced later in pregnancy, increased placental EV secretion may be a compensatory attempt to improve glucose uptake, with this effect regulated by the placental glucose monitoring enzyme, OGT. Our results here demonstrate that placental OGT is associated with both changes in circulating EV concentration (Fig. 4A) and with changes in glucose sensitivity (Fig. 4D–F), supporting the hypothesis that circulating EVs may contribute to the energy balance required for pregnancy, and to overall maternal health during this physiologically demanding time period.

## Discussion

Disruptions to maternal homeostatic regulation during pregnancy, such as those induced by chronic stress, are associated with adverse maternal health outcomes, including preterm birth, gestational diabetes, and preeclampsia^1,42–44^. The placenta plays a key role in homeostatic regulation by coordinating communication between maternal and fetal compartments, in part, through the production of extracellular vesicles (EVs)^45^. While studies have examined how physiological challenges in pregnancy affect fetal development, the role of EVs in maternal health has not been well examined. Our previous work identified placental O-glycosyl transferase (OGT) as a target of maternal stress, with reductions in OGT levels and activity, including modification of the OGT target, Annexin A1 (ANXA1), a protein involved in EV secretion^6,7^. OGT, a glucose-sensing enzyme, post-translationally O-glycosylates serine and threonine residues of hundreds of proteins^46–49^. The enzymatic activity of OGT is highly affected by glucose metabolism in a given cell, and changes to OGT function have been associated with metabolic disorders, including diabetes^50–54^.

Glucocorticoids play a significant role in the regulation of glucose homeostasis^55^. Here, we examined the hypothesis that placental OGT, which is reduced by maternal stress, impacts maternal homeostatic regulation during pregnancy via a change to circulating EV concentrations. EVs are lipid bound, nano-sized particles secreted by cells that carry nucleic acids, proteins, and other signaling factors, facilitating cell–cell and long-distance communication in the body, and have garnered growing interest as biological communicators and biomarkers of disease in recent years^22,28,45,56–59^. We evaluated the concentration, size, and ζ-potential of isolated circulating EVs in response to changes in metabolic regulators important for homeostasis during pregnancy. Where the size of an EV may provide information regarding the type of vesicle predominantly in circulation (e.g., exosome, microvesicle, macrovesicle), the ζ-potential, or surface charge, indicates particle stability in circulation, as well as its potential for uptake^45,60^. Dynamic changes in EV circulating concentrations, such as the three to fourfold increase in circulating EVs observed during pregnancy, suggest important biological functions related to homeostatic regulation important in health outcomes, especially in pregnancy^21,27,33,45,59,61^.

We first examined the influence of key metabolic factors that change in response to circadian and feeding rhythms on EV production. We found that over the circadian rhythm, as corticosterone increased, the concentration of EVs in circulation significantly decreased in non-pregnant animals. Similar observations were previously reported in samples from human patients, where increased EV concentration corresponded to decreased cortisol^62–64^. We also found that direct injection of metabolic factors, including corticosterone, glucose, or insulin, significantly and rapidly altered EV concentration. Indeed, others have shown changes to both EV production and diurnal variation of important metabolic functions in mice after deletion of secretory vesicle proteins^62–64^. Together, these data support a coordination of dynamic EV signals in homeostatic regulation.

Pregnancy is a metabolically demanding state, and control of homeostatic regulation is critical for maternal health^5,40,42,44,65–67^. The profound increase in circulating EV concentration found in pregnancy is attributed to placental EV secretion^31,37,45,68–70^. Indeed, placental EVs have been implicated in a number of pregnancy morbidities, and identified as key communicators in maternal pathophysiology^21,28,29,31,59,61,71^. To assess changes to the concentration of EVs during pregnancy, we utilized our established model of maternal stress as a perturbation. Similar to effects seen with a corticosterone injection, we found a persistent and significant reduction in EV concentration throughout pregnancy. These data support a lasting effect of stress to alter EV concentrations in circulation throughout pregnancy.

As EVs detected in circulation are contributed by most tissues in the body, assessing the effect of stress specifically on placental EV secretion required in vitro modeling. Therefore, we examined the effect of cortisol on EV secretion using human trophoblast-like BeWo cells to mimic the placental response to stress. Similar to effects following stress or corticosterone injection in vivo, EV secretion was significantly reduced following cortisol treatment of these cells. A decrease in EV concentration associated with stress has been observed in other model systems where exposure to stress or stress hormones resulted in decreased EV concentration^58,72,73^.

Most Eutherian animals become less glucose sensitive as pregnancy progresses^40^. Similarly, circulating EV concentrations continue to increase across pregnancy in many species^61,74^. Placental EVs were previously shown to mediate glucose uptake in skeletal myocytes in vitro, with differential outcomes based on whether the EVs were derived from normal glucose tolerant or gestational diabetes patients^31^. Further, EV concentrations were significantly increased in gestational diabetes pregnancies compared to normal pregnancy levels, suggesting again the potential for compensatory responses by the placenta to increase EV production to counter the rise in glucose^37^. As OGT is a glucose-sensing enzyme, we hypothesized that the increasing concentration of circulating EVs found in pregnancy may contribute to glucose sensitivity and uptake^41,52^. To determine the potential role of placental EVs important to maternal health, we examined changes in glucose tolerance in response to an acute elevation in systemic EVs. We administered EVs from pregnant dams into nonpregnant dams via tail vein injection, thereby doubling the concentration of EVs in circulation and mimicking circulating EVs during pregnancy. While we observed no significant differences, we found that doubling the concentration of EVs in circulation trended toward decreasing the intra-group variance, potentially indicating a synchronization of glucose uptake across tissues. A limitation of our experimental design is that pregnancy is a unique biological state and non-pregnant females may not be the best proxy for the action of EVs during pregnancy. However, as pregnancy EV concentrations are so profoundly high, it was not experimentally feasible to further elevate levels in the pregnancy state.

We next investigated the correlation between placental OGT and EV concentrations, important for glucose homeostasis, by directly targeting placental OGT levels. We utilized placental-specific transgenic targeting to produce litters which had variable OGT scores across dams, allowing us to assess changes in maternal circulating EV concentrations. We hypothesized that, similar to effects we found with maternal stress where both OGT and EVs were decreased by stress, directly reducing placental OGT would also impact EV concentrations. Indeed, we found that the total placental OGT score was significantly and positively correlated with the EV concentration in maternal circulation. Importantly, we established that this association is reflective of placental OGT levels, and not the number of placentae in the litter.

We next wanted to determine if this positive association between placental OGT levels and maternal EV concentration was predictive of maternal glucose tolerance and regulation across pregnancy. As expected, we detected an overall shift to the right in maternal glucose tolerance across pregnancy in the mice, reflective of the reduction in insulin sensitivity known to occur^40,75^. However, when we divided our dams into low and high OGT score groups, with high placental OGT levels associated with high EV concentration, the shift to the right in the glucose curve between E12.5 and E15.5 was reversed, while in the low placental OGT group it was not. These results suggest that the placental response to increasing glucose levels may be an increase in EVs to offset insulin insensitivity. Recent studies reported changes in maternal circulating EV concentration associated with gestational diabetes, where increased EVs were detected, supporting a potential compensatory attempt by the placenta to resolve a non-homeostatic state^31,37^. Based on our experiments, it is not clear if or where EVs are acting to alter glucose homeostasis (e.g., liver, skeletal muscle), but this could be the focus of future studies.

Our results demonstrate a potential novel relationship between placental OGT and the concentration of EVs in circulation during pregnancy. In our studies examining glucose tolerance by manipulating placental OGT using transgenic lines to vary total OGT between litters, we found that increasing EVs prevented the glucose insensitivity that occurs later in pregnancy. These data suggest a potential role of EVs in communicating glucose homeostasis, which is required for maintenance of both maternal and fetal health. It may be that the inability of the placenta to elevate EVs in circulation limits the signals necessary to coordinate or synchronize biological functions essential to balancing the energy demands of the mother and fetus; that the more EVs in circulation, the more equipped a system is to control glucose levels. There is evidence of this interplay between stress and glucose regulation in clinical studies, where a history of depression and trauma was associated with impaired glucose tolerance during pregnancy^42^. While many questions remain surrounding the function of EVs in maternal and fetal health, our current studies establish a potential role for placental OGT in maternal health during pregnancy. We highlight the importance of EV concentration in circulation as a key consideration in understanding how EVs regulate maternal homeostasis during pregnancy, and their potential role as detectable biomarkers in identifying health risks. Future studies could focus on unique EV cargo that are likely involved in the protein–protein targeting and signaling important for local tissue effects, the types of EVs produced by the placenta, and the specific mechanism by which EVs impact glucose uptake across various tissues^76–80^.

## Methods

### Animals

Mice used in these studies were bred in-house and were derived from a mixed background (C57Bl/6:I129) strain. Dams were between 10 and 16 weeks old. Virgin dams were used for all pregnancy experiments, and litters of 5–10 pups were included in analyses. To establish pregnancy, dams were paired with sires from 1900 to 0700 h. Embryonic day 0.5 (E0.5) was considered 1200 h on the day that a copulation plug was identified. Dams were evaluated on E12.5, E15.5, and E18.5. Unless otherwise specified, all collections were performed between 0700 and 1100 h.

Mice used to determine the effects of O-GlcNAc transferase (OGT) on extracellular vesicles (EVs) and glucose tolerance were double-heterozygous (Ogt^tm1Gwh^/J (X^OGT^/X^WT^); CYP19-Cre (P.Cre^+/−^)^7,81^. Dams were bred to either wildtype males (mixed background C57Bl/6:I129), or males which were hemizygous for OGT (X^OGT^/Y) and heterozygous for CYP19-Cre (P.Cre^+/−^) resulting in offspring representing all possible genotypes (X^WT^/X^WT^, X^OGT−/^X^WT^, X^OGT−^/X^OGT−^, X^WT^/Y, X^OGT−^/Y). Dams were euthanized at E12.5 (n = 10), E15.5 (n = 11), E18.5 (n = 8). Fetal tail DNA was isolated from each of the offspring and used for genotyping and calculating the OGT score. Based on previous findings, wildtype females (X^WT^/X^WT^) were assigned 2 points; wildtype males (X^WT^/Y) and X^OGT−/^X^WT^ females were assigned 1 point; and knock-out males and females (X^OGT−^/Y, X^OGT−^/ X^OGT−^) were assigned 0 points.

All animals used in this study were euthanized using a precision vaporizer with an induction chamber and waste gas scavenger in which isoflurane was administered in 2.5% O_2_ for 1 min. Decapitation was performed after cardiac puncture to ensure euthanasia. All procedures in this study were approved by the University of Maryland Baltimore Institutional Animal Care and Use Committee. All procedures were conducted in accordance with the National Institutes of Health Guide for the Care and Use of Laboratory Animals. Reporting of animal experiments is done in accordance with ARRIVE guidelines.

### Extracellular vesicle (EV) isolation and characterization

Blood was collected via cardiac puncture using 26 G syringes (Becton Dickinson). Dipotassium EDTA coated tubes were used for blood collection (Becton Dickinson Microtainer™). Blood was spun at 1300 rcf for 10 min at 4 °C to separate plasma. EVs were isolated from 250 μL of blood plasma, as previously reported^82^. Plasma was mixed with 500 μL of freshly filtered (0.22 μm pore size filter, Sigma) phosphate-buffered saline (PBS). The plasma-saline mixture was centrifuged at 2000 rcf for 30 min at 4 °C, followed by centrifugation at 12,000 rcf for 45 min at 4 °C to pellet cell debris. EVs were immediately isolated from the resulting supernatant via size exclusion chromatography using the IZON qEVoriginal 70 nm columns in the IZON Automated Fraction Collector (Izon Science). Protein LoBind Tubes (Eppendorf™)were used for collection. Fractions 2 and 3 were combined and stored at – 80 °C until the sample was characterized, at which time samples were thawed on ice.

The concentration, size, and ζ-potential of the isolated EVs were measured using a ZetaView^®^ BASIC Nanoparticle Tracking Analysis Microscope (Particle Metrix)^60^. EVs were diluted in freshly filtered water for a final concentration in the range of 10^7^ particles/mL (~ 200 particles per frame). The size and concentration of particles were measured by scanning 11 cell positions, with 30 frames per position, over 2 cycles. ζ-potential was measured across 11 positions. The sensitivity for video acquisition was set to 80, and the shutter speed to 100. ZetaView^®^ software 8.05.14 was used to analyze all videos, with minimum brightness set to 20, minimum area to 10, and maximum area to 1000.

### Probing mediators of extracellular vesicle concentration

Circadian collections in non-pregnant dams were done at 0700 (n = 4), 1100 (n = 5), 1500 (n = 4), and 1900 (n = 4) hours. To measure corticosterone levels, 10 μL of tail blood was collected and mixed with 5 μL of 50 mM EDTA (Sigma). Blood was kept on ice until centrifugation for 10 min at 5600 rcf at 4 °C. Plasma was collected and stored at − 80 °C until analysis. Cardiac puncture was performed to collect blood for EV isolation, as described above.

Non-pregnant dams were separated into two groups: vehicle (saline, n = 5) and corticosterone (Cayman Chemical, 300 μg/kg, n = 5). At 0800, dams were given an intraperitoneal injection based on their assigned group. After 30 min, blood was collected via cardiac puncture and used for EV isolation, as above.

Non-pregnant dams were separated into five groups: non-injected (n = 4), vehicle (saline, n = 5), fast (fasted 0800 h to 1400 h, saline intraperitoneal injection, n = 5), glucose (Sigma, fasted 0800 h to 1400 h, 3 mg glucose/g body weight in saline intraperitoneal injection, n = 6), insulin (Humulin, 0.8 mU insulin/g body weight in saline intraperitoneal injection, n = 5). Blood from control animals was collected at 1400 h. Vehicle, insulin, fasted, and glucose groups received respective intraperitoneal injections, and blood was collected after 2 h.

### Corticosterone radioimmune assay

Corticosterone levels were determined using a corticosterone double antibody radioimmunoassay (RIA, MP Biomedicals), according to the kit instructions and as previously described^83,84^. Briefly, plasma was diluted 1:200 using the steroid diluent included in the kit. ^125^I corticosterone was added to each sample, followed by anti-corticosterone. Tubes were incubated for 2 h at room temperature. Precipitant was added to each tube, and tubes were spun at 2500 rpm for 15 min at 4 °C. Liquid was aspirated from the tubes, and the resulting pellet was measured using a gamma counter (Perkin Elmer, Wizard^2^).

### BeWo b30 cells

Human choriocarcinoma trophoblastic cells, BeWo b30 (AcceGen, passage 7) were cultured in low glucose (1000 mg/L) Dulbecco’s modified Eagle’s medium (DMEM, Sigma) supplemented with 10% charcoal-stripped fetal bovine serum (FBS, ThermoFisher) and 1% penicillin/streptomycin (P/S, ThermoFisher) at 37 °C in 5% CO_2_^70^. Cells were plated at a density of 2.5 million cells per T-175 flask for 6 days, with media changes every 3 days. On day 6, cells were washed with sterile PBS, and grown in DMEM supplemented with 10% exosome depleted FBS (Gibco) and 1% P/S. Flasks were divided into two groups and treated with either vehicle (0.1% ethanol, n = 5) or 500 ng/mL cortisol (n = 5). After 3 days, media was collected and processed for EV isolation.

To isolate EVs, media was spun at 2500 rcf for 30 min at 4 °C. The resulting supernatant was spun at 12,000 rcf for 45 min, followed by two rounds of ultracentrifugation at 100,000 rcf for 100 min in a swing bucket rotor. As above, pelleted EVs were resuspended in sterile filtered PBS and characterized using the ZetaView. Non-conditioned media (n = 8) was processed in the same way.

### Early pregnancy stress

Pregnant dams assigned to the stress group underwent multimodal stress for seven days (0.5–6.5). As previously reported, non-habituating, painless stressors were grouped into three categories: odor, auditory, and tactile^7,85–90^. Odor stressors included ethanol (Sigma), fox odor (Sigma), or puma odor (Sigma), where dams were exposed to an open tube of the odor. Fox and puma odor were diluted 1:5000 in mineral oil (Sigma). Auditory stressors included owl screech and white noise, where the noise was played in the room at constant decibels (95–105 dB). Tactile stressors included wire mesh, wet bedding, and restraint. In the wire mesh stressor, chicken wire (Fencer Wire) cut to fit on the cage bottom was placed on top of the cage bedding. In wet bedding, 300 mL of water was poured onto the bottom of the cage, and in restraint, mice were put into a 50 mL Falcon Tube (Fisher) for the duration of stress. Dams were exposed to stressors from two categories from 0900 h until 1100 h, on a semi-random schedule. On E12.5 (n = 7), E15.5 (n = 6), and E18.5 (n = 7), dams were sacrificed, and blood was collected for EV isolation. Control non-pregnant dams (n = 9) were similarly sacrificed, and control pregnant dams were sacrificed on E12.5 (n = 8), E15.5 (n = 10), and E18.5 (n = 8). The number of pups in each litter was recorded. Stressors did not affect maternal food consumption, weight gain, litter size, or sex ratio^7,85–90^.

### EVs to alter glucose tolerance

Non-pregnant dams were fasted starting at 0800 h. After 6 h of fasting, dams were given a tail vein injection of either vehicle (PBS, n = 5) or E18.5 EVs at a final concentration of 5 × 10^9^ particles/g body weight in PBS (n = 6). EVs used in these experiments were collected from E18.5 control X^WT^/X^WT^ dams, as described above. A third group of animals received no injection (n = 7). After 15 min, dams were challenged with an intraperitoneal injection of glucose (3 mg/g body weight) and glucose readings were measured at 0, 30, 60, and 120 min timepoints using the Contour Next Blood Glucose Monitoring System (Bayer Co, Germany), as previously reported^91^.

### Glucose tolerance test

Glucose tolerance tests were performed, as previously reported^91^. Pregnant dams (X^OGT−/^X^WT^) were fasted beginning at 0800 h on E12.5 (n = 11), E15.5 (n = 10), and E18.5 (n = 8). A cohort of non-pregnant dams (n = 7) was also used. After 6 h of fasting, dams received an intraperitoneal injection of glucose in saline (3 mg/g body weight). Glucose readings were collected by tail blood at 0, 30, 60, and 120 min timepoints using the Contour Next Blood Glucose Monitoring System (Bayer Co, Germany). Following the glucose tolerance test, dams were sacrificed, fetal tails were collected to evaluate OGT score.

### Statistical analysis

Sample size, mean, standard error of mean (SEM), and statistical significance are reported in the text and figure legends. GraphPad Prism was used to apply appropriate statistical analyses. For experiments of two groups, an unpaired t test was utilized. For experiments with three or more groups, analysis of variance (ANOVA) testing with Tukey post-hoc tests were used, with a significance set at *p* < 0.05. Metabolic testing was analyzed using ANOVA with repeated-measures corrections and Tukey post-hoc tests. Pearson correlations were used to analyze EV characteristics as a function of OGT score. 95% Confidence Intervals are shown for correlation data. Data are presented as mean ± SEM. GraphPad Prism and BioRender were used to generate figures.

## Supplementary Information

Supplementary Figures.

## Data Availability

The data analyzed in this study are available from the corresponding author upon request.
