# Supplementary material for: Extracellular vesicles are dynamic regulators of maternal glucose homeostasis during pregnancy
Source: Sci Rep. 2023 Mar 20;13:4568. doi: 10.1038/s41598-023-31425-x (PMC10027885; doi:10.1038/s41598-023-31425-x)
Supplement: Supplementary file 1 — Supplementary Figures. [file 41598_2023_31425_MOESM1_ESM.pdf]

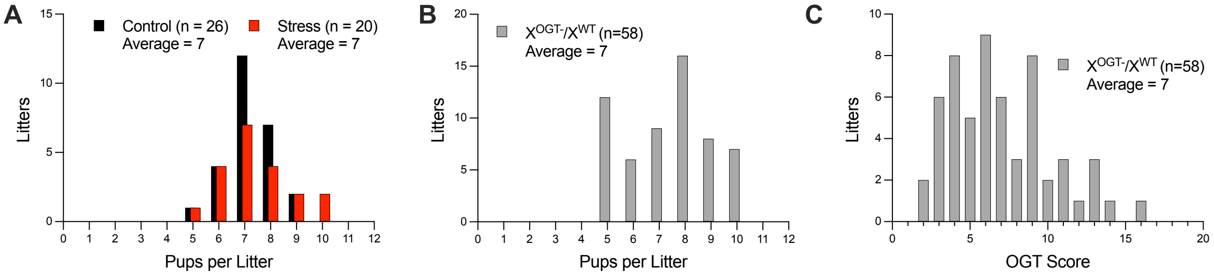

Supplementary Figure 1: Litter size is not affected by stress or genotype. (**A**) Control litters (black bars, n=26) had an average of 7 pups per litter. Stress litters (red bars, n=20) had an average of 7 pups per litter. (**B**) Litters from X^{OGT-}/X^{WT} dams (n=58) had an average of 7 pups per litter. (**C**) The average OGT score for litters from X^{OGT-}/X^{WT} was 7.

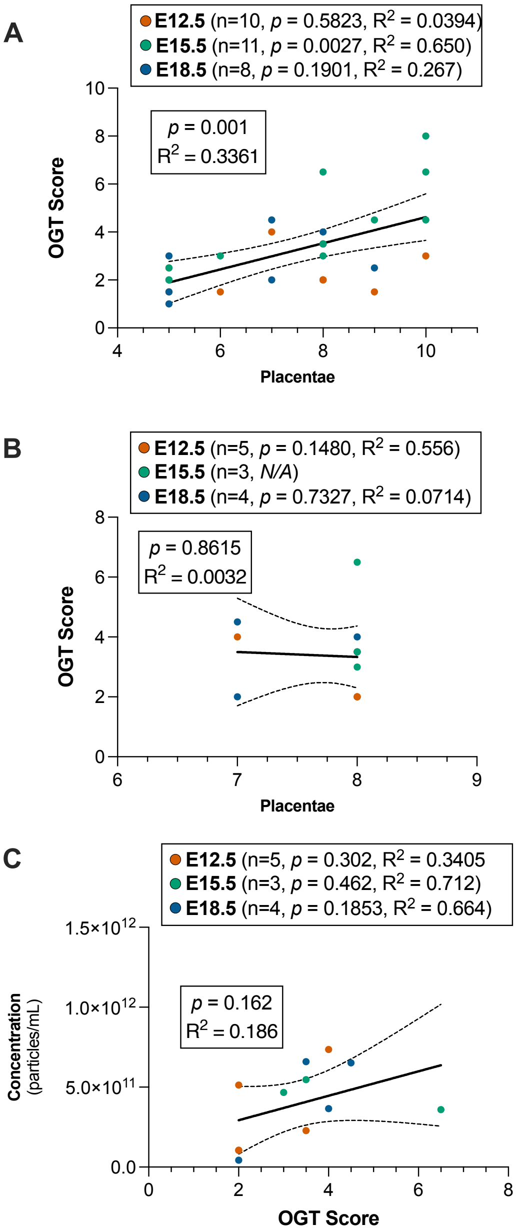

Supplementary Figure 2: OGT score affects EVs, independent of number of placentae. We sought to explore the relationship between OGT score and number of placentae. (**A**) The number of placentae is significantly correlated to the OGT score (Pearson correlation, $F_{1,27}$=13.67, p = 0.001, R^2 = 0.3361). This is not surprising, as more placentae increase the possibility for more copies of OGT. Note that there are three identical datapoints for E12.5, with 8 placentae resulting in an OGT score of 2, an identical data point for E12.5 and E18.5 with 5 placentae resulting in an OGT score of 1.5, a second identical data point for E12.5 and E18.5 with 8 placentae resulting in an OGT score of 3.5, and two identical datapoints for E15.5 with 5 placentae resulting in an OGT score of 2. (**B**) Including only litters of 7-8 pups, we see that the OGT score ranges from 2-7 (Pearson correlation, $F_{1,10}$=0.032, p = 0.8615, R^2 = 0.0032). Note that there are three identical data points for E12.5 with 8 placentae resulting in an OGT score of 2, and an identical data point for E12.5 and E18.5 with 8 placentae resulting in an OGT score of 3.5. (**C**) Including only litters of 7-8 pups, we see a positive correlation between OGT score and concentration of particles in circulation (Pearson correlation, $F_{1,10}$=2.279, p = 0.162, R^2 = 0.1856). Note that there is a nearly identical data point for E12.5, where an OGT score of 2 resulted in an EV concentration of 1.04 x 10^{11} and 1.05x10^{11}. 95% CI are shown as dotted lines above and below the trend line for (**A**,**B**,**C**).

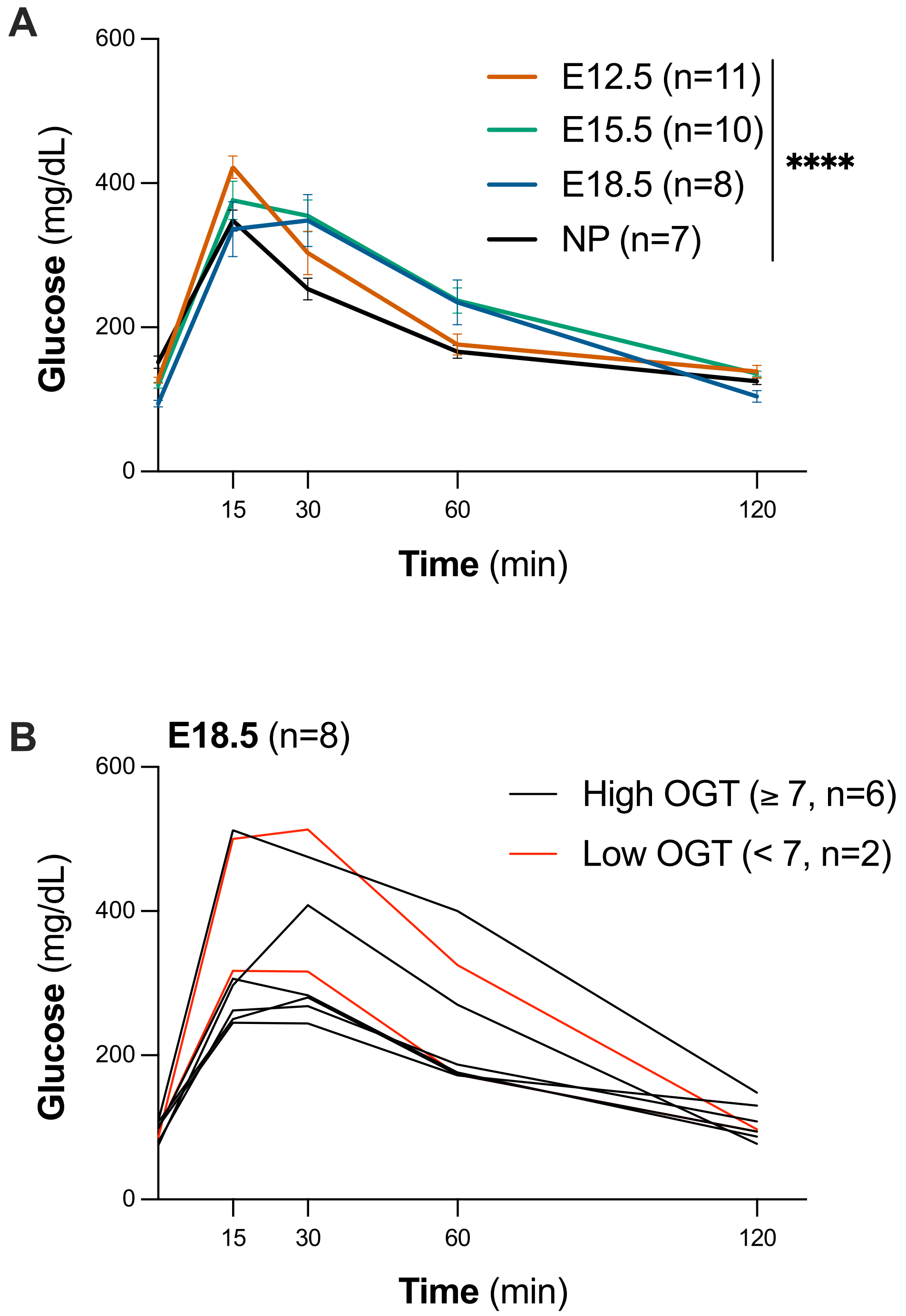

Supplementary Figure 3: OGT in the placenta correlates with maternal glucose sensitivity. (**A**) Glucose tolerance for non-pregnant (n=7, NP, black), E12.5 (n=11, orange), E15.5 (n=10, green), and E18.5 (n=8, blue) dams. Non-pregnant, E12.5, and E15.5 curves are repeated from Figure 4D, where we observed significant variation in glucose sensitivity across gestational days (two-way ANOVA, main effect of time, $F_{2.078, 66.49}=210.2$, $p < 0.0001$, main effect of group, $F_{3,32}=1.116$, $p = 0.3571$, time*group interaction, $F_{12,128}=4.887$, $p < 0.0001$). (**B**) Glucose tolerance for individual dams on E18.5 broken up by OGT score, where low was considered < 7 (n=2, red lines), and high ≥ 7 (n=6, black lines).
